# Lack of Racial Survival Differences in Metastatic Prostate Cancer in National Cancer Data Base (NCDB): A Different Finding Compared to Non-metastatic Disease

**DOI:** 10.3389/fonc.2020.533070

**Published:** 2020-09-18

**Authors:** Toms Vengaloor Thomas, Xiaoshan Z. Gordy, Seth T. Lirette, Ashley A. Albert, David P. Gordy, Srinivasan Vijayakumar, Vani Vijayakumar

**Affiliations:** ^1^Department of Radiation Oncology, University of Mississippi Medical Center, Jackson, MS, United States; ^2^Department of Health Sciences, University of Mississippi Medical Center, Jackson, MS, United States; ^3^Department of Data Science, University of Mississippi Medical Center, Jackson, MS, United States; ^4^Department of Radiology, University of Mississippi Medical Center, Jackson, MS, United States

**Keywords:** prostate cancer, metastasic, racial and ethnic differences, African American (AA) men, white men, survival outcomes

## Abstract

**Background:** Inconsistent findings have been reported in the literature regarding racial differences in survival outcomes between African American and white patients with metastatic prostate cancer (mPCa). The current study utilized a national database to determine whether racial differences exist among the target population to address this inconsistency.

**Methods:** This study retrospectively reviewed prostate cancer (PCa) patient data (*N* = 1,319,225) from the National Cancer Database (NCDB). The data were divided into three groupings based on the metastatic status: (1) no metastasis (*N* = 318,291), (2) bone metastasis (*N* = 29,639), and (3) metastases to locations other than bone, such as brain, liver, or lung (*N* = 952). Survival probabilities of African American and white PCa patients with bone metastasis were examined through parametric proportional hazards Weibull models and Bayesian survival analysis. These results were compared to patients with no metastasis or other types of metastases.

**Results:** No statistically supported racial disparities were observed for African American and white men with bone metastasis (*p* = 0.885). Similarly, there were no racial disparities in survival for those men suffering from other metastases (liver, lung, or brain). However, racial disparities in survival were observed among the two racial groups with non-metastatic PCa (*p* < 0.001) or when metastasis status was not taken into account (*p* < 0.001). The Bayesian analysis corroborates the finding.

**Conclusion:** This research supports our previous findings and shows that there are no racial differences in survival outcomes between African American and white patients with mPCa. In contrast, racial disparities in the survival outcome continue to exist among non-metastatic PCa patients. Further research is warranted to explain this difference.

## Introduction

Prostate cancer (PCa) was the most frequently diagnosed cancer in over 100 countries in 2018, and the fifth leading cause of cancer-related deaths in men, according to Global Cancer Statistics 2018 (1). In the United States, PCa accounts for 20% of new cancer diagnoses in men (2). Race is a well-studied risk factor for PCa (3–7). It is widely recognized that when compared to white men, African American men are more likely to develop PCa, tend to be diagnosed at a younger age, exhibit higher prostate-specific antigen (PSA) levels, and demonstrate more advanced or metastatic disease at diagnosis (8–12). Besides, African American men are four times more likely to develop metastatic PCa (mPCa) than white men and more than twice as likely to die from PCa (13–19). It is of vital importance to continue research on racial disparity of PCa to better understand the disease and improve the survival outcomes of African American men.

The existing literature has consistently reported that racial survival differences exist between African American and white patients with localized PCa (20–24). However, inconsistent findings have been observed regarding the survival outcomes of African Americans and whites with mPCa. Some studies reported that African American men had poorer survival outcomes than white men (25–27); while other studies detected no differences (6, 28–33); and still others found that African American men might have better overall survival than white men (34, 35).

To address the inconsistency in the literature and to further investigate mPCa, we conducted a pilot study that examined African American and white PCa patients with bone metastasis at an academic medical center (36). The results revealed that when treatments were equal, no differences in survival outcomes were observed between African American and white men (36). Based on the pilot study, we hypothesized that mPCa might have different behavior from non-metastatic PCa in different races. Even though our initial study included 12-year retrospective data, the sample size was relatively small. Therefore, the current study utilized the National Cancer Database (NCDB), which contained nationwide cancer data collected from over 1,500 facilities and provided a sample size that was larger than the majority of existing studies. We aimed to evaluate any potential racial differences in the survival outcomes of African American and white patients with mPCa.

## Methods

The National Cancer Database (NCDB) is a nationwide, hospital-based registry that consists of data from patients who received care at cancer centers accredited by the American College of Surgeons Commission on Cancer (CoC). It is estimated to capture ~70% of all patients newly diagnosed with cancer in the United States (37). The CoC's NCDB and the accredited facilities participating in the NCDB are the sources of the de-identified data used in this study. However, the CoC has not verified and are not responsible for the statistical validity or conclusions derived by the authors of this study. Because the NCDB contains de-identified patient data, this study did not meet our institutional review board's (IRB) criteria for human subject's research, and thus approval and review from IRB was not indicated. The use of de-identified data in this study complied with the terms specified in the NCDB Participant User File Data Use Agreement. Individual patients, hospitals, and healthcare providers were not identified.

The prostate cancer-specific dataset provided by NCDB was used to assess our primary research objectives. Of the available 1,380,357 men in the original data, 61,132 were excluded for non-white/African American race, leaving 1,319,225 in the initial analysis dataset. Of these, 348,882 had metastasis data available. Patients were divided into three metastases groupings: (1) no metastasis (*N* = 318,291) (2) bone metastasis, including bone and other metastases (*N* = 29,639), and (3) metastases to locations other than bone, such as brain, liver, or lung metastasis (*N* = 952). Full data were used in analyses where metastasis status was not considered.

### Statistical Analysis

Means with standard deviations and counts with percentages were compiled for baseline comparisons between African Americans and whites within each metastasis category and in the overall cohort. All henceforth described models were adjusted in the respective regression models for baseline age, treatment facility type and location, insurance status, tumor grade, census tract median income, percent with a high school degree, and Charlson-Deyo comorbidity score.

Adjusted failure time curves were constructed from parametric proportional hazards models using Weibull distributions to model time-to-death first disregarding metastasis status and then stratified by metastasis category. From these curves, we estimated the yearly survival probabilities.

In order to incorporate as much prior information as possible, especially the wealth of studies that have shown African Americans have lower long-term survival than whites, Bayesian survival analysis was performed. Similar pooled and stratified Weibull models were constructed, as described above, with the exception being that now we place a highly informative Cauchy (log (2), 0.5) prior on the log-hazard for the African Americans main effect with all other parameters having non-informative priors. This model, particularly the log (2) location parameter, allows us to use the previously published information that African American men have twice the hazard of death compared to whites. This fully informative prior was compared to a non-informative prior. From our primary model, posterior histograms of estimated hazard ratios were plotted. Posterior means, medians, and 95% credible intervals were further compiled. All Bayesian posterior checks were performed to assure proper model convergence. All analyses were completed with Stata v15.1 (StataCorp, College Station, TX).

## Results

As shown in Table 1, the overall cohort had a mean age of 65.3 and a median follow-up time of 5.3 years. Those who were not missing metastasis data had a mean age of 65.1 and a median follow-up time of 3.4 years. Those with metastasis were slightly older in both whites and African Americans. The majority (>75%) of primary treatments occurred at comprehensive community cancer programs or academic/research programs. Most were either privately insured or on Medicare. Tumor grades were most likely to be moderately differentiated or poorly differentiated, except in the bone metastasis category, where a high percentage of tumor grades were not determined. African Americans tended to live in areas with lower incomes, and a higher percentage of residents without a high school diploma compared to whites. Charlson-Deyo scores were roughly evenly distributed across races.

**Table 1:** Sample participant characteristics (AA: African Americans).

	**Overall Cohort**		**No Mets**		**With Bonemets**		**W/O Bonemets But W/ Other Mets**	
	**Whites (*N* = 1126439) 100%**	**AA (*N* = 192786) 100%**	**Whites (*N* = 272253) 24%**	**AA (*N* = 46038) 24%**	**Whites (*N* = 23885) 2%**	**AA (*N* = 5754)3%**	**Whites (*N* = 774) 0.07%**	**AA (*N* = 178) 0.09%**
**Age**	65.69 (9.04)	62.95 (9.00)	63.36 (8.07)	60.65 (8.05)	72.37 (11.26)	67.26 (10.76)	73.81 (11.17)	68.48 (11.34)
**Facility Type**								
Community Cancer	96906 (9%)	16220 (8%)	20781 (8%)	3439 (7%)	2909 (12%)	591 (10%)	94 (12%)	12 (7%)
Comp. Community Cancer	506080 (45%)	69767 (36%)	116838 (43%)	17044 (37%)	10924 (46%)	1789 (31%)	347 (45%)	70 (39%)
Academic/Research	410471 (36%)	81701 (42%)	106580 (39%)	19825 (43%)	7750 (32%)	2651 (46%)	261 (34%)	66 (37%)
Integrated Network Cancer	112360 (10%)	24831 (13%)	27883 (10%)	5661 (12%)	2289 (10%)	719 (13%)	71 (9%)	30 (17%)
**Facility Location**								
New England	76367 (7%)	5811 (3%)	16723 (6%)	1255 (3%)	1702 (7%)	160 (3%)	58 (8%)	2 (1%)
Middle Atlantic	166994 (15%)	31388 (16%)	38012 (14%)	6440 (14%)	3685 (15%)	951 (17%)	114 (15%)	24 (13%)
South Atlantic	220574 (20%)	68892 (36%)	51424 (19%)	16765 (36%)	4181 (18%)	1921 (33%)	151 (20%)	68 (38%)
East North Central	207417 (18%)	33095 (17%)	50181 (18%)	7523 (16%)	4560 (19%)	1072 (19%)	138 (18%)	31 (17%)
East South Central	78513 (7%)	18605 (10%)	22818 (8%)	5237 (11%)	1237 (5%)	479 (8%)	52 (7%)	15 (8%)
West North Central	106585 (9%)	6398 (3%)	28572 (11%)	1713 (4%)	2137 (9%)	204 (4%)	69 (9%)	7 (4%)
West South Central	74622 (7%)	17517 (9%)	18158 (7%)	4741 (10%)	1650 (7%)	592 (10%)	54 (7%)	20 (11%)
Mountain	58328 (5%)	1754 (1%)	15899 (6%)	460 (1%)	1493 (6%)	69 (1%)	41 (5%)	2 (1%)
Pacific	136417 (12%)	9059 (5%)	30295 (11%)	1835 (4%)	3227 (14%)	302 (5%)	96 (12%)	9 (5%)
**Insurance Status**								
Not Insured	14608 (1%)	7667 (4%)	3511 (1%)	1837 (4%)	992 (4%)	589 (10%)	22 (3%)	15 (8%)
Private	523569 (46%)	87927 (46%)	150053 (55%)	24677 (54%)	5711 (24%)	1247 (22%)	161 (21%)	45 (25%)
Medicaid	18046 (2%)	12704 (7%)	4643 (2%)	2920 (6%)	1225 (5%)	884 (15%)	30 (4%)	20 (11%)
Medicare	528362 (47%)	74137 (38%)	106384 (39%)	14310 (31%)	15293 (64%)	2843 (49%)	540 (70%)	90 (51%)
Other Government	17102 (2%)	5586 (3%)	4418 (2%)	1594 (3%)	295 (1%)	78 (1%)	8 (1%)	1 (1%)
Not Known	24752 (2%)	4765 (2%)	3244 (1%)	700 (2%)	369 (2%)	113 (2%)	13 (2%)	7 (4%)
**Tumor Grade**								
Well Differentiated	58873 (5%)	10447 (5%)	24847 (9%)	3996 (9%)	131 (1%)	34 (1%)	6 (1%)	2 (1%)
Moderately Differentiated	487134 (43%)	79174 (41%)	116611 (43%)	20025 (43%)	1080 (5%)	330 (6%)	42 (5%)	10 (6%)
Poorly Differentiated	520743 (46%)	89895 (47%)	116231 (43%)	19460 (42%)	12675 (53%)	2969 (52%)	330 (43%)	85 (48%)
Undifferentiated	4622 (0%)	936 (0%)	502 (0%)	106 (0%)	233 (1%)	58 (1%)	20 (3%)	3 (2%)
Not Determined	55067 (5%)	12334 (6%)	14062 (5%)	2451 (5%)	9766 (41%)	2363 (41%)	376 (49%)	78 (44%)
**Median Income**								
< $38,000	136548 (12%)	73408 (38%)	31307 (12%)	16329 (36%)	3485 (15%)	2592 (45%)	117 (15%)	77 (44%)
$38,000–$47,999	247497 (22%)	42791 (22%)	58625 (22%)	10112 (22%)	5634 (24%)	1308 (23%)	160 (21%)	42 (24%)
$48,000–$62,999	311494 (28%)	39225 (21%)	76404 (28%)	9860 (22%)	6805 (29%)	1084 (19%)	215 (28%)	34 (19%)
$63,000 +	421435 (38%)	35719 (19%)	105208 (39%)	9558 (21%)	7845 (33%)	744 (13%)	281 (36%)	24 (14%)
**% No High School Degree**								
21% +	133585 (12%)	60332 (32%)	30528 (11%)	13300 (29%)	3656 (15%)	2148 (37%)	120 (16%)	61 (34%)
13–20.9%	248797 (22%)	67336 (35%)	59044 (22%)	15765 (34%)	5571 (23%)	2035 (36%)	178 (23%)	79 (45%)
7–12.9%	388118 (35%)	43769 (23%)	94350 (35%)	11267 (25%)	8110 (34%)	1107 (19%)	284 (37%)	21 (12%)
<7%	347143 (31%)	19833 (10%)	87752 (32%)	5553 (12%)	6449 (27%)	442 (8%)	191 (25%)	16 (9%)
**Charlson-Deyo Score**								
0	951240 (84%)	154067 (80%)	224968 (83%)	35389 (77%)	17949 (75%)	4319 (75%)	583 (75%)	130 (73%)
1	146034 (13%)	31506 (16%)	40190 (15%)	8836 (19%)	3938 (16%)	906 (16%)	135 (17%)	31 (17%)
2	22923 (2%)	5091 (3%)	5593 (2%)	1275 (3%)	1379 (6%)	351 (6%)	38 (5%)	8 (4%)
3	6242 (1%)	2122 (1%)	1502 (1%)	538 (1%)	619 (3%)	178 (3%)	18 (2%)	9 (5%)

*Shown are means and standard deviations for age. All other statistics are raw counts and percentages*.

**Figure 1:**
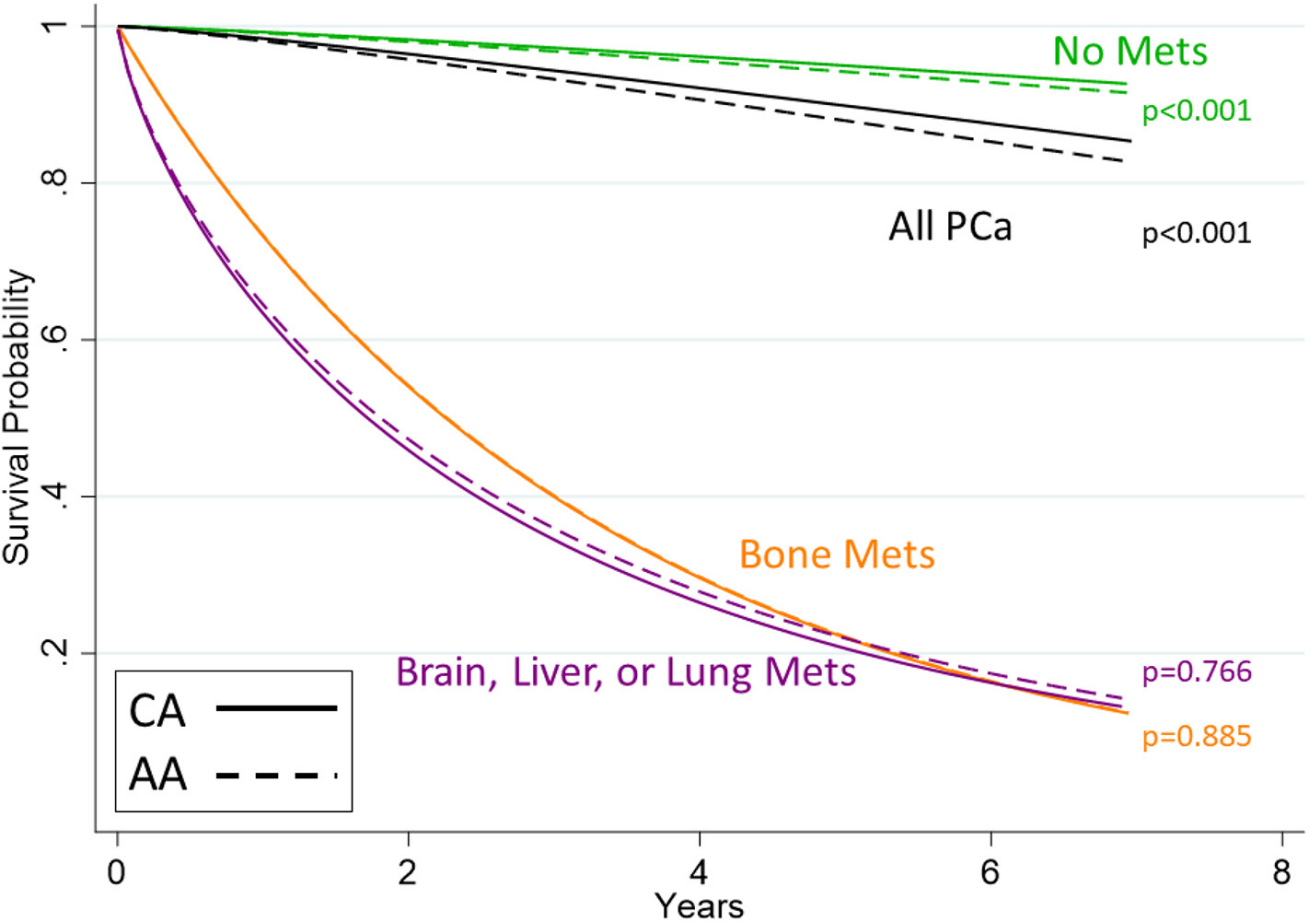
Survival curves for men with prostate cancer stratified by metastasis category. Shown are curves estimated from Weibull models. Whites are shown with solid lines and African Americans with dashed lines. The black line shows all PCa diagnoses, regardless of metastases status. Of note is that the lines representing racial distinctions are indistinguishable for the bone metastasis group (orange) since the African American effect contributes virtually nothing to the hazard of death (*p* = 0.885) nor for metastasis other than bone (purple, *p* = 0.766). This null effect was not observed in the group with no metastasis (*p* < 0.001, green lines).

Adjusted racial differences were observed when not taking metastasis status into account (*p* < 0.001). Similar differences were seen for those men having no metastasis (*p* < 0.001). These translated to 5 and 7-year survival probabilities of 0.95 vs. 0.94 and 0.93 vs. 0.91 (whites vs. African Americans), respectively. No statistically supported racial disparities were observed for those men with bone metastasis (*p* = 0.885), which translates to a five-year survival probability of 0.22 and seven-year survival probability of 0.12, regardless of race. Similar results were observed for those men suffering from other metastases. These results can be seen in Table 2 as well as Figure 1.

**Table 2:** Predicted probabilities of yearly survival by metastasis type (AA: African Americans).

		**No Mets (*****N*** **=** **318291)**		**Bone Mets (*****N*** **=** **29639)**		**Other Mets (*****N*** **=** **952)**	
		***p*****-value for race diff**. **<** **0.001**		***p*****-value for race diff**. **=** **0.885**		***p*****-value for race diff**. **=** **0.766**	
		**Whites**	**AA**	**Whites**	**AA**	**Whites**	**AA**
Year	1	0.99	0.99	0.72	0.72	0.63	0.64
	2	0.98	0.98	0.54	0.54	0.46	0.47
	3	0.97	0.97	0.40	0.40	0.34	0.36
	4	0.96	0.96	0.29	0.29	0.26	0.28
	5	0.95	0.94	0.22	0.22	0.2	0.22
	6	0.94	0.93	0.16	0.16	0.16	0.17
	7	0.93	0.91	0.12	0.12	0.13	0.14

*Probabilities are derived from parametric Weibull models. Although these differences are not largely substantial, even for groups with statistically supported differences, the further extended on the time horizon, the further the disparities will become apparent in the “No Mets” and “Other Mets” categories. These probabilities are adjusted for baseline age, treatment facility type and location, insurance status, tumor grade, census tract median income, and percent with a high school degree, and Charlson-Deyo comorbidity score*.

**Figure 2:**
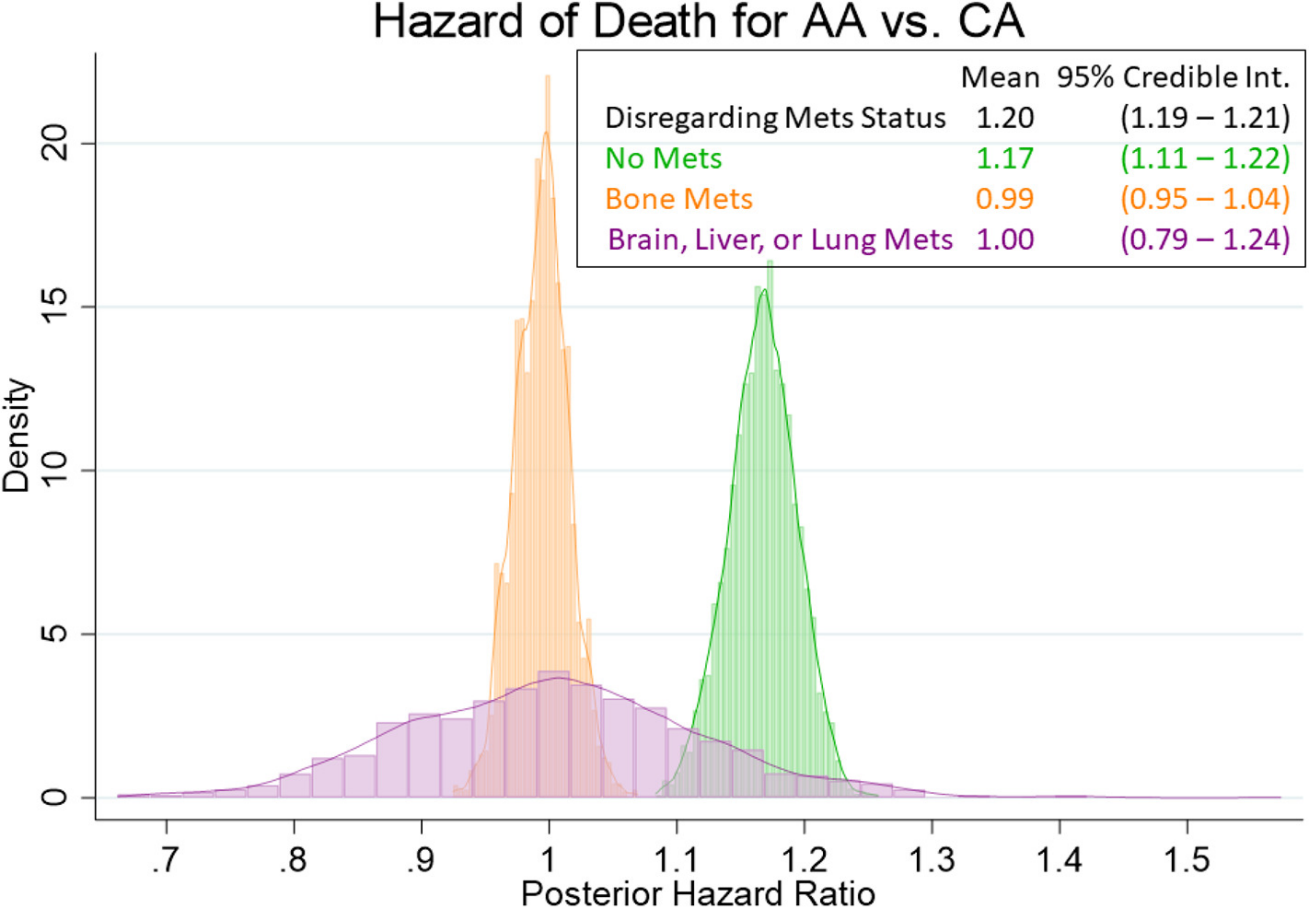
Posterior distributions of hazard ratios for the African American main effect. Posterior hazard ratios come from Bayesian Weibull models with informative Cauchy (log (2), 0.5) priors on the African American main effect. The histograms (coupled with analogous estimates) show that there is a demonstrable (if not modest) racial effect on the hazard of death for those men without metastasis (HR = 1.17), as well as with the overall cohort (HR = 1.20). However, the racial disparities are virtually nonexistent for those mean with bone metastasis (HR = 0.99) or with brain, liver, or lung metastasis (HR = 1.00).

Our Bayesian analysis corroborates the same information as the frequentist analysis presented in the preceding paragraph. As shown in Figure 2, even with priors assuming double the hazard of death for African Americans vs. whites, the data pulled the posterior hazard ratios more closely to 1. There was still a modest, but meaningful effect for those men without metastasis [HR = 1.17; 95% Cred. Int. (1.11–1.22)] as well as when we disregard metastasis status [HR = 1.20; 95% Cred. Int. (1.19–1.21)]. Once again, this effect disappeared for men with bone metastasis [HR = 0.99; 95% Cred. Int. (0.95–1.04)] and for men with metastasis other than bone [HR = 1.00; 95% Cred. Int. (0.79–1.24)].

## Discussion

Our study found that there is no disparity in survival for African American and white patients with metastatic prostate cancer, even though a disparity exists for patients with non-metastatic prostate cancer. This study is the biggest study revealing an absence of a racial disparity in the survival outcome for metastatic prostate cancer. Our results are in agreement with studies that analyzed patients with metastatic prostate cancer—either androgen-sensitive metastatic prostate cancer (mPCa) or Castrate Resistant Prostate Cancer (mCRPC). However, there are some conflicting data in the available literature as well.

In the case of androgen-sensitive mPCa, Sassani et al. retrospectively reviewed 681 metastatic prostate cancer patients who received ADT as monotherapy in the Kaiser Permanente Southern California Cancer Registry between January 2003 and December 2006 (38, 39). They found that among these patients, African American men had a 34% lower risk of biochemical failure compared with white men (HR = 0.66, *p* = 0.03) (38, 39). McLeod et al. conducted an exploratory analysis of patient data from a double-blind, randomized, multicenter trial which evaluated the use of combined androgen blockade in 813 patients with mPCa and found that there were no significant differences in the survival outcomes among African Americans and whites (33). Gordy et al. recently reported in a retrospective study from our intuition an absence of a racial disparity in the treatment outcomes for metastatic prostate cancer patients (36). Thompson et al. analyzed patients enrolled into Southwest Oncology Group Study 8,894 randomized phase III trial that compared orchiectomy with or without flutamide in men with metastatic prostate cancer to determine if ethnicity was an independent predictor of survival (26, 40). Upon analysis of the data from 288 African American and 975 white patients in the trial, they found African American men had a worse survival (HR = 1.23, *p* = 0.018), after adjusting for confounding variables (26).

Regarding mCRPC, the controversy continues as to whether a racial disparity in outcome exists. Thatai et al. retrospectively reviewed 145 patients with androgen-independent prostate cancer enrolled in clinical trials at Wayne State University between 1991 and 2001. Even though there was no difference by race in the distribution of patient and disease characteristics, the race was found to be the only significant factor predicting time to PSA progression (4.6 months in whites vs. 2.3 months in African Americans, *p* = 0.02) (31). However, there was no difference in overall survival by race (31). However, when Halabi et al. retrospectively reviewed the data of men with Metastatic Castrate Resistant Prostate Cancer (mCRPC) enrolled in 9 phase III trials treated with Docetaxel containing regimens (34, 41), they found that African American patients were younger, and had worse performance status, higher testosterone, and prostate-specific antigen, and lower hemoglobin than whites. Despite these differences, both races had an equivalent median survival of 21 months. Multivariate analysis revealed that African American men had a 19% decreased risk of death compared to white men (HR = 0.81, 0.72 to 0.91, *p* < 0.001) (34, 41). In a different study by Halabi et al. analyzed patients with mCRPC enrolled in 8 Cancer and Leukemia Group B (CALGB) trials and reported that there is no significant difference in survival based on race (42). In a retrospective analysis of 1,902 mCRPC patients treated with Sipuleucel-T, African American men were found to have a significantly better median overall survival compared to white men (35.2 vs. 29.9 months, CI: 0.68–0.97, *p* = 0.03) (43).

McNamara et al. conducted a retrospective evaluation of chemotherapy-naïve mCRPC patients treated with Abiraterone or enzalutamide from a Veterans Health Administration (VHA) database that included 2,123 white and 787 African American mCRPC patients (35). Even though African American men had higher numbers of comorbidities, they still had better OS compared to white men (HR = 0.826; 95%CI [0.732-0.933]) (35). “Abi Race” (NCT01940276) is a prospective, multicenter study of African American and white patients with mCRPC treated with abiraterone acetate and prednisone, evaluating racial disparities in the outcomes (44). When George et al. reported the initial results of this prospective study, they found the primary endpoint radiographic progression-free survival (rPFS) was equal among the races (16.8 months) (44). Interestingly, African American men had more significant and more durable PSA progression-free survival, which was their secondary endpoint (28). Matzkin et al., Fowler et al. and Bergan et al. have all reported the absence of racial disparities in the treatment outcomes for mCRPC patients (29, 32, 45). These results should be analyzed acknowledging the disparities in the clinical trial participation among the races.

Our study agrees with the general literature regarding the presence of racial disparities in the outcome for localized prostate cancer (no metastasis group). There is evidence to suggest that African American men are disproportionately affected by prostate cancer (46, 47), and it has been reported in other countries other than the United States as well (48). There is some evidence to suggest that African American men have a genetically different cancer (16, 21, 31, 49, 50). However, other authors argue that there are no established genetic differences in PCa among African Americans and whites and that the disparities are due to differences in access to care (51). African American men tend to have poor access to screening for prostate cancer (52), and African American men tend to have a lower use of follow-through care even after having an elevated PSA identified during screening (53). Some authors have concluded that disparities continue to exist between African Americans and whites regarding treatments of localized PCa (54, 55), including African American men are less likely to receive definitive treatments (54, 56–58); less likely to undergo prostatectomy or pelvic node dissection during prostatectomy and are more likely to have complications during the operation and in the post-operative period (55, 59, 60).

There is some recent research suggesting that access to care is the causative factor leading to the racial disparities in the outcome. These authors argue that no racial disparities exist in the outcome if there is equal access to care, based on studies of patients who received care through Veterans Affairs hospital system or in a clinical trial, where similar treatments are guaranteed (6, 61). Krimphove et al. reported that when access to care, treatment, and cancer characteristics are accounted for, African American men were found to have even better OS as compared to white men (62).

Our study presents a difference in racial disparities in the outcome between metastatic and non-metastatic prostate cancer patients, which is hard to explain. One possible explanation is the treatment modality. Metastatic patients are treated with systemic therapy only, and that was mainly Androgen Deprivation Treatment (ADT) until recently. There is some evidence to suggest that African American men have higher testosterone and androgen receptor expression compared to white men (21, 63, 64). These might be contributing to a better response to ADT, which in turn might nullify other socio-economic factors contributing to the disparities in the outcome. Another possible explanation is the wide availability and relatively inexpensive nature of ADT, leading to equal opportunities for treatment regardless of race and socioeconomic status. Localized PCa is a very heterogeneous disease with multiple significantly different treatment options, which might lead to disparities in the outcome (65). In contrast, mPCa is a relatively homogeneous disease with only a few treatment options and thus have less chance of racial disparity in the treatment outcomes. Further research is warranted to determine why there is an absence of racial disparities in the mPCa and its implications.

The primary limitation of our study is the retrospective nature of the NCDB database which accounts for patients receiving care only in Commission on Cancer (CoC) accredited facilities. This is a hospital-based registry and there is possibility that we might be missing more number of patients, unlike in a population-based registry (37). We did not evaluate the treatment outcomes based on the treatment options. The cause of death or cancer-specific survival was not available due to the nature of the NCDB database (37).

## Conclusions

Current NCDB review aimed to validate our previously published results of an absence of racial disparities in the survival outcome among PCa patients with bone metastases. This research supports our previous finding and reveals that there are no racial differences in survival outcomes between African American and white patients with metastatic prostate cancer (bone or other metastases) in a national database. In contrast, racial disparities in the outcome continue to exist among non-metastatic PCa patients. Further Research is warranted find the cause of this difference.

## Data Availability Statement

The datasets generated for this study are available on request to the corresponding author.

## Ethics Statement

Ethical review and approval was not required for the study on human participants in accordance with the local legislation and institutional requirements. Written informed consent for participation was not required for this study in accordance with the national legislation and the institutional requirements.

## Author Contributions

TV and XG are the lead authors who share first authorship. SL participated in data analysis, table/figure creation, and data interpretation. AA contributed to manuscript writing and review. DG contributed to manuscript writing and review. SV and VV are the senior authors who came up with the original idea for this research and guided the research and writing process. All authors contributed to the article and approved the submitted version.

## Conflict of Interest

The authors declare that the research was conducted in the absence of any commercial or financial relationships that could be construed as a potential conflict of interest.
